# Nonlinear dielectric spectroscopy biosensor for SARS-CoV-2 detection

**DOI:** 10.1038/s41598-022-20961-7

**Published:** 2022-10-12

**Authors:** Ali Talebipour, Amir Hosein Ghannad, Elham Sharifi, Morteza Pirzadeh, Hamed Hasanzadeh Moghadam, Mehrdad Saviz, Majid Badieirostami, Parham Karimi Reikandeh, Hamid Mobasheri, Reza Faraji-Dana

**Affiliations:** 1 Department of Biomedical Engineering, Amirkabir University of Technology (Tehran Polytechnic), Tehran, Iran; 2 School of Electrical and Computer Engineering, College of Engineering, University of Tehran, Tehran, Iran; 3 Biophysics Workgroup, University Consortium of Covid-19, Tehran, Iran; 4 Department of Electrical Engineering, Amirkabir University of Technology (Tehran Polytechnic), Tehran, Iran; 5 Laboratory of Membrane Biophysics and Macromolecules, Institute of Biochemistry and Biophysics, University of Tehran, Tehran, Iran; 6 Center of Excellence on Applied Electromagnetic Systems, University of Tehran, Tehran, Iran

**Keywords:** Viral infection, Biomedical engineering, Biotechnology

## Abstract

The coronavirus disease caused by the SARS-CoV-2 virus has affected people worldwide for more than two years. Here we present a new diagnostic method based on nonlinear dielectric spectroscopy to detect the presence of the SARS-CoV-2 virus in swab samples. A known current is injected into the virus sample suspension, and the biomarker is the third harmonic detected in the power spectrum of the recorded signal. Computational modeling of harmonic production supports the hypothesis of ion channels (the E-protein) with nonlinear current–voltage characteristics being present on the virus envelope as a possible origin of harmonics. The developed system is able to distinguish between positive and negative samples with 5–10 dBc (decibels relative to the carrier) higher third harmonic ratios in positive samples, in agreement with the computational estimation. Our early results demonstrate that this method can detect the virus in solution. This is the first time harmonic signatures are used to detect SARS-CoV-2 in swab samples.

## Introduction

It has been more than two years since the outbreak of the SARS-CoV-2 virus in 2019 and the announcement of the Covid-19 Pandemic. Although nowadays vaccines are being used globally to harness the epidemic, their efficiency is not ideal, and the pandemic is yet taking victims. Since then, various biomarkers have been used to diagnose the disease. The virus genome is the most potent biomarker used in most point-of-care (POC) diagnostic kits. There are several routine techniques being used to detect the virus, including; polymerase chain reaction (PCR), qRT-PCR, RT-LAMP, CRISPR, and NGS (next-generation genome sequencing)^1^. The World Health Organization (WHO) has recommended PCR to be considered as the golden standard method for coronavirus detection and COVID-19 diagnosis. Though this method has sufficient sensitivity, it comes with roughly ten percent false-negative or false-positive results^2^. Furthermore, this laboratory-based technique is time-consuming, expensive, and requires advanced tools and skilled clinical lab technicians^3^. Due to the fast spread of the virus and the consequent high infectivity and mortality rates, it is vital to innovate fast-response, low-cost, and user-friendly SARS-CoV-2 detection techniques^2^. Therefore, alternative rapid POC diagnostic kits are extremely desired.

Biosensors are simple, real-time, and effective devices capable of detecting various infectious diseases^4^. A biosensor is usually composed of a sensing agent that interacts with the biological molecules to produce a meaningful signal for detection. This signal is known as a biomarker for the presence of pathogens. The monoclonal antibodies, nucleic acids, glycan, lectin, and enzymes are the most frequently used sensing agents in biosensors. The response of the biosensor, i.e., the signal produced from the pathogen interacting with the receptor, is converted into a measurable signal by a transducer. Accordingly, the presence of the pathogen can be revealed after thorough processing of the recorded signals^4^. Different types of biosensors based on electrochemical, optical, and microfluidics approaches have been developed for the COVID-19 diagnosis, and some even made their way to the market. Viral proteins, viral genome, and antibodies specific to viral components generated by the host immune system are the three most used biomarkers in these biosensors^1^. Nevertheless, to harness the widespread of different lethal strains of viruses across the globe, research and development of yet even more efficient, sensitive, accurate, instant, economic, and, if possible real-time biosensors are extremely desired.

Physically, coronavirus is a spherical particle with a diameter of 100–160 nm enclosed in an envelope lipid bilayer, in which the membrane (M), envelope (E), and spike (S) structural proteins are embedded^1,5^. Recent studies have indicated that several SARS-CoV-2 proteins, including E, ORF3a, and ORF8a, can self-assemble into oligomers and generate ion channels (ICs)^6^. Kern et al. strongly suggest that 3a proteins are non-selective cation channels^7^. Numerous studies have established the relevance of the E protein and protein 3a as fundamental pro-inflammatory SARS-CoV virulence factors. They have suggested that these proteins act as ion channel proteins^5^. The unique distribution of different transmembrane proteins in the SARS-CoV-2 demonstrates a more complex structure than integrated solid-state semiconductors. The virus possesses a capacitive membrane formed by arrays of parallel lipid molecules. This lipid membrane is formed in a polar and highly dynamic water medium and holds different proteins, including rectifying ion-channel forming proteins, ORF3a.

Power Spectrum Analysis has been a major tool to detect particular characteristics of the event(s), including amplitudes, energy ratio, frequency, noise, correlation, probability density, cumulative distribution function, etc. occurred in the recorded signal from different sources through the Fast Fourier Transform (FFT)^8^. The relevant statistical signal analysis aspects have been applied to detect single nanoparticles and biological molecules such as yeast ribosomes floating in fluids exposed to laser beams^9^. Furthermore, Dielectric spectroscopy has been utilized to measure the size and distribution of oil-in-water particles in fluids^10^. However, we think nonlinear dielectric spectroscopy has been originally applied to detect a biological organism, yeast, by Woodwards and Kell^11^. The results of theoretical, statistical and practical studies of biological and non-biological material conducted here is consistent with the relevant aspects considered in the mentioned literatures and well approves the capability and credibility of our method towards detection of viruses in solution.

Nonlinear dielectric spectroscopy (NLDS) has been shown to be capable of detecting some bacteria in suspension based on the harmonic pattern observed in the Fourier transform of their corresponding voltage signal^11,12^. However, to our best knowledge, no study has yet used this method to detect the presence of virus in the solution. Also, most ion channels on the membrane do not have ohmic behaviour and instead show a nonlinear I-V relation which consequently results in harmonics generation.

Especially because of nonlinear ion channels on the SARS-CoV-2 viral particles, we hypothesize that power spectrum harmonics can be used as a signature for the electronic detection of coronavirus. The sputum samples from patients were used to detect the presence of the virus through a novel biosensor based on the harmonic changes in the Fourier spectrum of the recorded voltage. By detecting the harmonic content in a patient's sample, we were able to develop new diagnostic criteria for the early detection of SARS-CoV-2. The virus detecting biosensor constructed here does not require any labeling scheme, is simple, inexpensive, non-invasive, and easy to use by patients, and can be further advanced to respond in real-time.

## Materials and methods

### Preparation of patient samples

The samples therein were throat swabs taken from patients and each kept in two separate vials in viral transport medium (VTM). One of the two vials was used for the RT-PCR test, and the result was labeled as positive or negative accordingly. The samples were loaded in our tailored airtight water-proof recording chamber, and remained tightly closed all throughout the experiments in BSL-2 labs. A virus-free VTM was used as the reference in all experiments conducted in this study. The samples were de-identified at the clinical laboratory prior to handing over for electrical experiments, with no numbers or labels (except for the positive or negative PCR results), and were numbered by the second hand operator. Electrical measurements described herein, were performed on the samples and the samples were safely discarded. All protocols of the clinical experiments were approved by the Research Center for Clinical Virology of Tehran University of Medical Sciences according to the obtained informed consent of all subjects and/or their legal committee under the Ethical License of IR.TUMS.VCR.REC.1399.599, issued in compliance with relevant laws and institutional guidelines in accordance with the ethical standards of the Declaration of Helsinki, by the Biomedical Research Ethics Committee of the Tehran University of Medical Sciences, Tehran, Iran.

### Nonlinear dielectric spectroscopy technique

**Figure 1:**
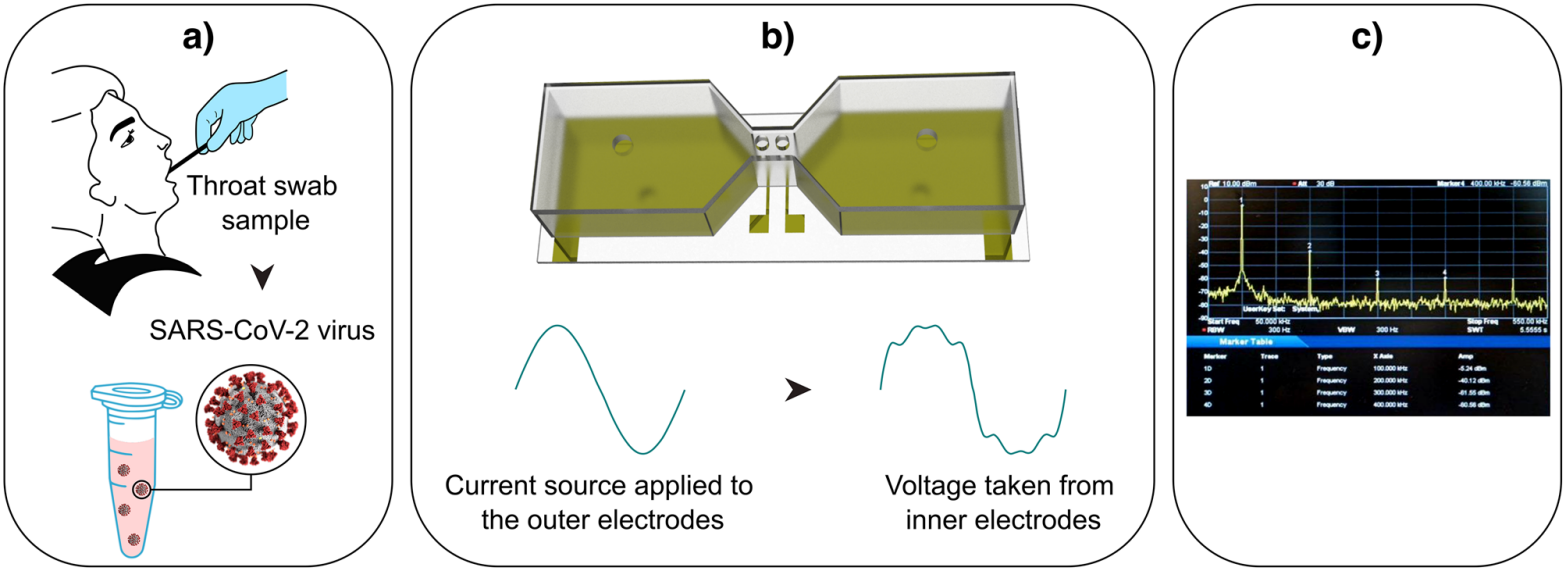
Different stages of the virus detector NLDS-biosensor. (**a**) Virus collection and sample preparation in the VTM solution, (**b**) schematic diagram of airtight biosensor chamber and NLDS generated signal, (**c**) typical NLDS power spectrum.

The NLDS technique was used to apply a single frequency sinusoidal current/voltage excitation to a bio-suspension solution loaded in the recording chamber of the biosensor. The power spectrum of the recorded voltage signals from the bio-suspension at different current levels was then calculated. The harmonics pattern observed in the power spectrum was further analyzed and interpreted in terms of membrane surface proteins. It should be noted that the electrodes were washed three times before each test with 70% ethanol, followed by pure deionized water. Figure 1 shows the complete picture of the workflow proposed in this diagnostic method.

Here, we aim to propose an innovative detection method for SARS-CoV-2 through a coherent combination of theoretical and experimental approaches. First, we theoretically estimate the power spectrum obtained from a virus solution using a mathematical model for the nonlinear flow of ion currents on the membrane surface. Second, the power spectrum of the virus-containing VTM solution is measured experimentally. Finally, the theoretical and experimental results are compared against each other to check for the possible existence of the SARS-CoV-2. Also, Statistical analyses were performed using GraphPad Prism version 9.3.1 and drawings were performed using Inkscape version 1.1 and GIMP version 2.10.28.

### SARS-CoV-2 membrane

The SARS-CoV-2 virus consists of a lipid membrane with a thickness of 5–7 nm^13,14^ containing spike and channel-forming proteins. The virus genome encodes three ion channels (E, 8a, and 3a). The E protein is the smallest of the major structural proteins in the virus. The 8a protein has been found in the membrane of the mitochondria as well as the endoplasmic reticulum, though it has not yet been established whether it forms an ion channel^5^. However, the 3a protein (also present in SARS-CoV-1) forms an ion channel and is involved in virus release, inflammasome activation, and eventual cell death^7^. Accordingly, we studied the activity of the 3a protein, the main ion channel of the virus, and modeled its I-V relation.

### Mathematical modeling of ion currents through SARS-CoV-2 membrane

**Figure 2:**
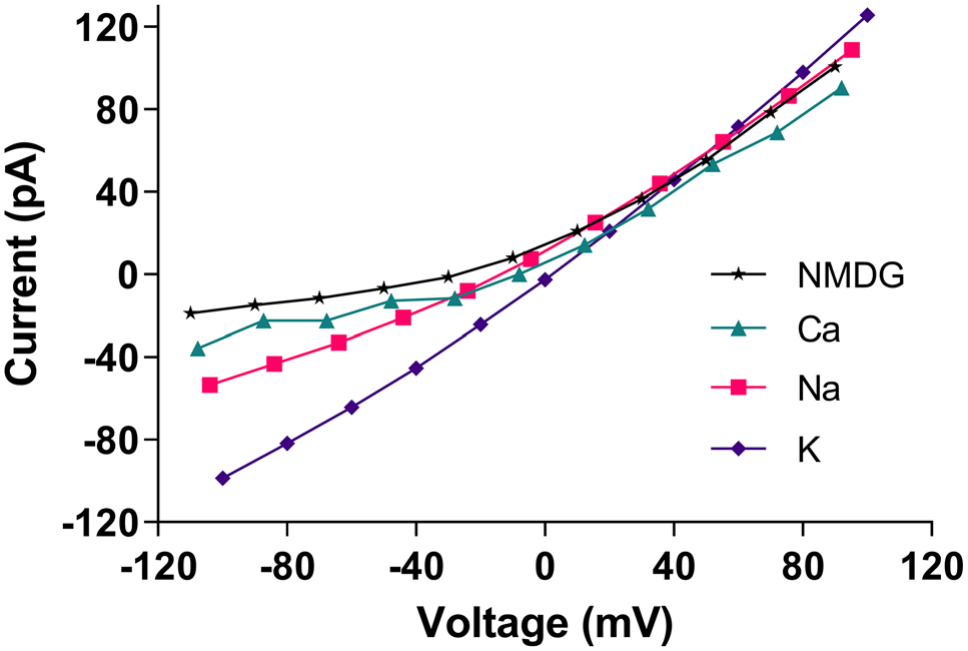
Current–voltage (conductance) diagram of 3a ion channel according to Kern et al.^7^. Drawings were made using Inkscape version 1.1 and GIMP version 2.10.28 (http://www.gimp.org, http://www.inkscape.org).

According to a study reported by Kern et al.^7^, the only membrane ion channel examined in the virus is the 3a channel. In this section, we use the current–voltage diagrams obtained from the study of SARS-CoV-2 protein 3a in a nanodisk of phosphatidylcholine lipids based on the currents recorded across excised patches pulled from proteoliposome blisters^7^ to develop a mathematical model of the nonlinear channel response. Figure 2 shows the current–voltage diagram of the 3a channel.

To obtain the mathematical model for the ionic currents of the virus membrane, the data for each ion type was fitted by a third-order polynomial with R^2^ > 0.99, and the current ${I}_{x}$ was calculated as a function of the membrane voltage. It is worth mentioning that gating of these channels has not been reported and therefore not included in this model.

By assuming a capacitance for the membrane, Eq. (1). Models the relationship between the voltage and the current passing through the virus membrane:

1 $$\begin{array}{ll} {C\frac{dV}{{dt}} = - I_{{\text{mem }}} = - \sum I_{x},\quad x = K,Na,Ca,NMDG} \\ {I_{x} = \alpha_{3x} V^{3} + \alpha_{2x} V^{2} + \alpha_{1x} V + \alpha_{0x} } \\ \end{array}$$

where ${I}_{x}$ represents ion current (µA), V, membrane voltage (mV), α_1-n_, current coefficient of individual ions, and x is indexing K, Na, Ca, and NMDG ions. Further, C = C_m_.A, where C_m_ is the membrane capacitance per unit area $\left(1 \; \upmu \text{F}/{\text{cm}}^{2}\right)$ and A the cell surface area. The current coefficients for each ion were worked out through curve fitting and are listed in Table 1.

**Table 1:** Current coefficients listed in Eq. (1) for different ions.

α_0x_	α_1x_	α_2x_	α_3x_	ion
− 2.305	1.12	1.603 × 10^–3^	− 1.646 × 10^–6^	K
11.46	0.8315	2.047 × 10^–3^	− 2.888 × 10^–7^	Na
8.15	0.6522	2.713 × 10^–3^	4.659 × 10^–6^	Ca
15.42	0.6479	3.288 × 10^–3^	6.063 × 10^–6^	NMDG

### Volume-conductor theory to simulate the virus-containing solution

The voltage across the virus membrane was calculated by volume-conductor theory using a mathematical model for the membrane. Here in five steps, we describe how the recorded nonlinear voltage on the electrodes related to ion channel’s nonlinear currents.

**Figure 3:**
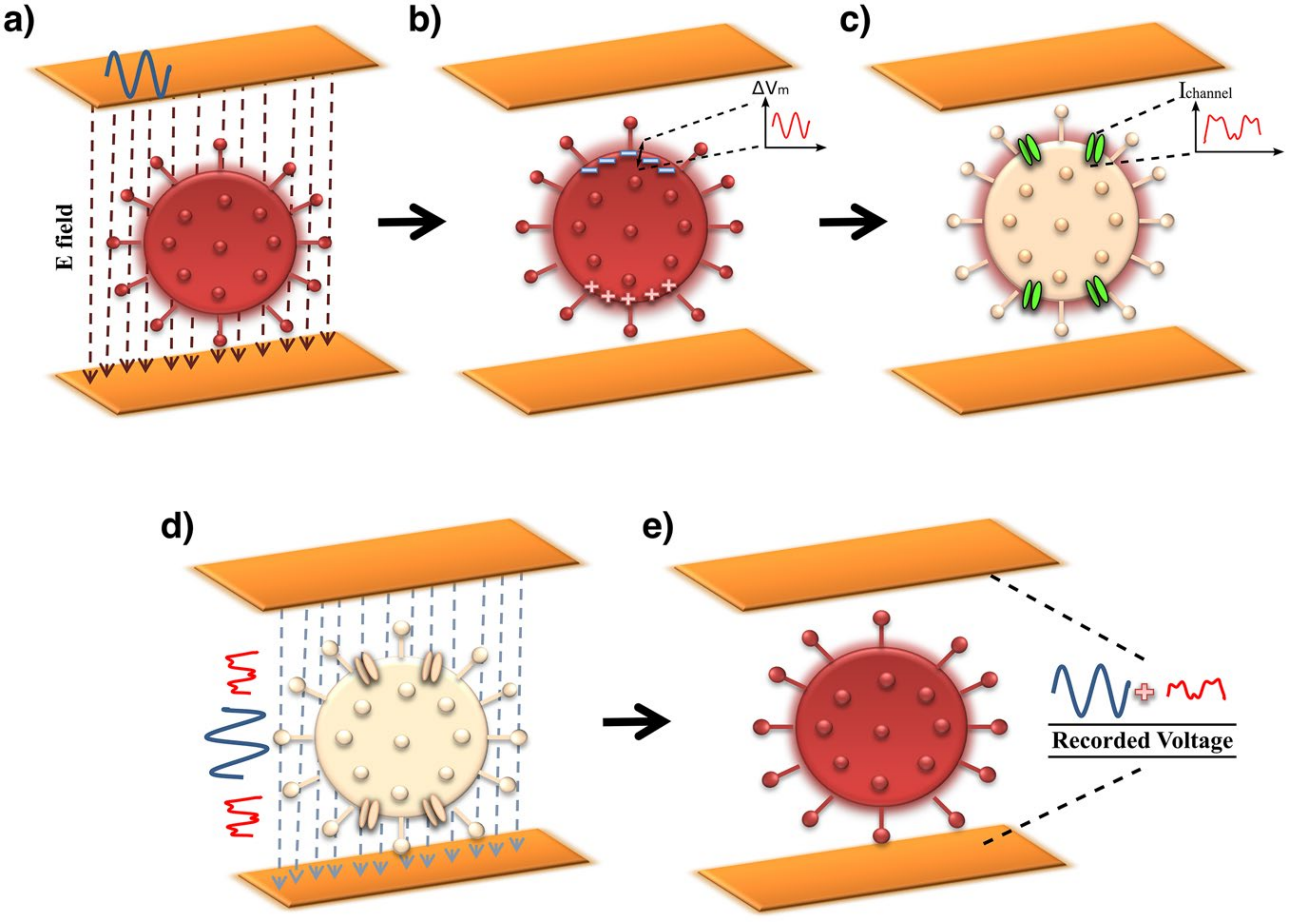
Schematic of a virus solution simulated by volume-conductor theory. (**a**) The sinusoidal voltage applied to the electrodes. (**b**) The SARS-CoV-2 viruses polarized along the field between the two electrodes. (**c**) The ion channels activated and generated nonlinear currents. (**d**) The linear and nonlinear parts of the current now flow through the solution. (**e**) Nonlinear currents are sensed at the electrode sites as a nonlinear voltage which ends up with adding odd harmonics to the power spectrum.
Step 2: In the presence of this electric field, positive and negative ions move in the solution. The Accumulation of ions in the non-conductive membrane site leads to the creation of a potential difference across the membrane, the maximum induced membrane voltage $\left( {\Delta V_{m} } \right)$ can be found by simple relations such as Eq. (2) as reported by Grosse et al.^15^:
  2 $$\Delta V_{m} = \frac{3}{4}D_{virus} \times E\;$$
  where $E$ is the field applied by the impedance meter (Fig. 3b).
Step 3: This potential difference across the membrane leads to the activation of ion channels. Due to the activity of the channels and the movement of ions through them, a nonlinear current is generated. Thereafter, the membrane channels are modeled as dipole currents^16^. Simulation of the membrane voltage caused by the virus-containing solution was calculated in MATLAB software.
  The detailed mathematical model of ion currents is presented in “Mathematical modeling of ion currents through SARS-CoV-2 membrane” section. The membrane voltage then defines the ion channel currents through Eq. (1) (see Fig. 3c).
Step 4: This nonlinear current flows through homogenous and linear volume conduction space (solution) and will receive at the electrode site (Fig. 3d).
Step 5: Finally, we measure the nonlinear voltage between two electrodes. This voltage is related to ion channel currents with volume conduction theory. This theory described more in the rest of the paper. This voltage is nonlinear because the ion channel current is nonlinear and this leads to the presence of harmonics in the power spectrum (Fig. 3e).

Step 1: When a sinusoidal voltage (V) is applied to parallel electrodes, a uniform electric field will be created between them, $E=V/d$; where d is the distance between two electrodes (Fig. 3a).

According to the volume-conductor theory, it is possible to calculate the potential recorded on the sensing electrodes using a quasi-static version of Maxwell's Equations. The volume-conductor theory relates the recorded potential to the current dipole on the membrane through Eq. (3),

3 $$V_{lead} = \sum {\vec{\mathbf{m}}} \cdot {\vec{\mathbf{c}}} = \smallint \left( {{\vec{\mathbf{J}}}_{tot} dv} \right) \cdot {\vec{\mathbf{c}}}$$

where $\overrightarrow{\mathbf{m}}$ represents the dipole vector, and $\overrightarrow{\mathbf{c}}$ is the lead vector depending on the electrode distance and the geometry.

Application of the electric field induces a voltage across the membrane and causes depolarization and hyperpolarization (Fig. 3b). The sum of the ionic currents caused by the voltage induction on the virus membrane is considered as the dipole current's total vector,

4 $${\vec{\mathbf{J}}}_{tot} = {\vec{\mathbf{J}}}_{dep} - {\vec{\mathbf{J}}}_{hyp}$$

where ${\overrightarrow{\mathbf{J}}}_{dep}$ and ${\overrightarrow{\mathbf{J}}}_{hyp}$ represent the membrane current density. These currents are calculated by substituting the induced depolarization and hyperpolarization voltages on the membrane in Eq. (1). Therefore, the voltage received at the electrode can be calculated by using Eqs. (1–4) in the right order. The Fourier transform of this voltage provides the power spectrum, including the desired harmonic pattern.

## Experimental characterization

### Electrode design

**Figure 4:**
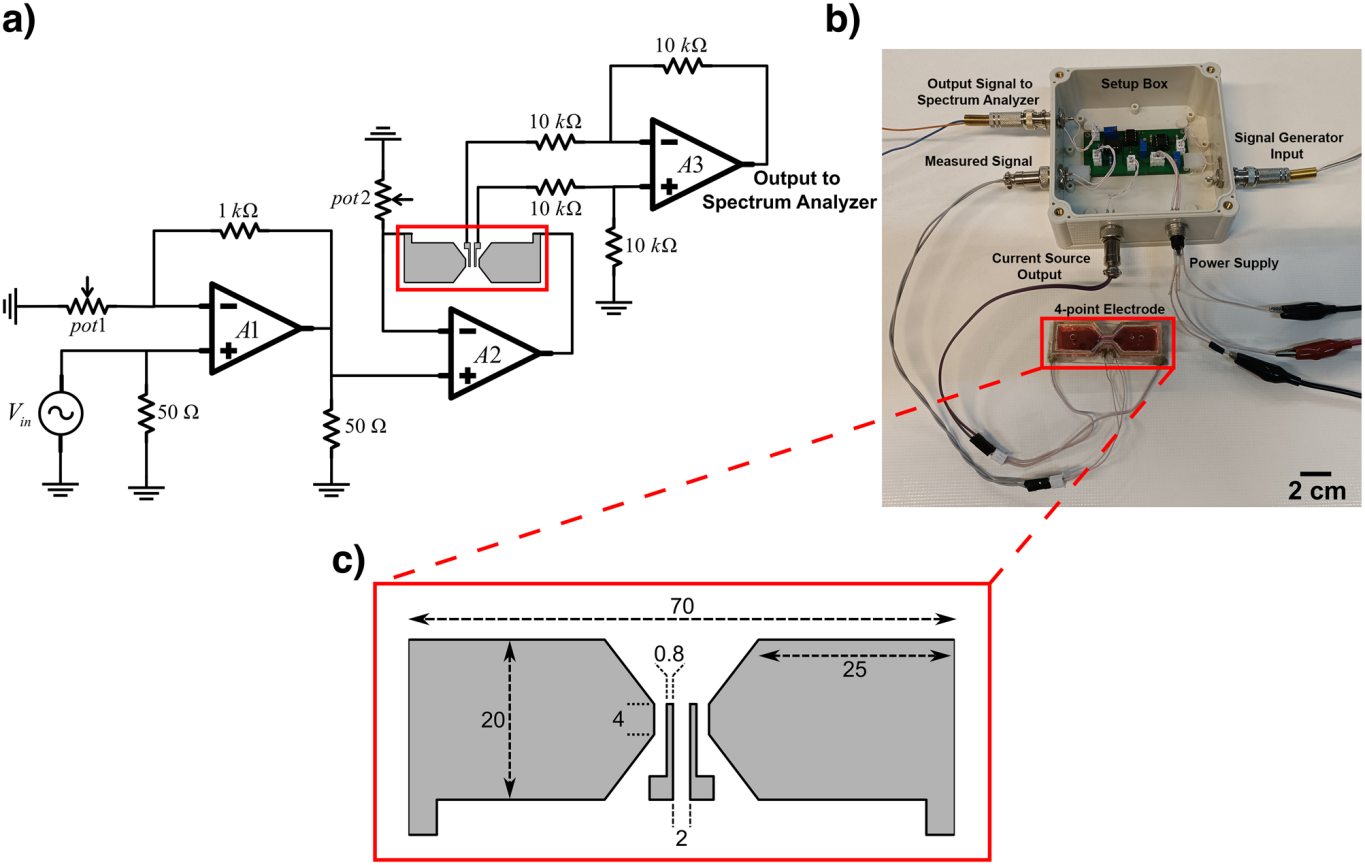
The NLDS biosensor (**a**) electronic deriving circuit, (**b**) measurement setup and (**c**) detailed drawing of the biosensor configuration showing the electrodes in gray and the size of different elements and recording chamber in millimeters.

Conventional dielectric spectroscopy techniques use two-, three-, or four-electrode systems to stimulate and record signals. Here, we used a four-electrode system in an especially designed chamber with a total volume of about 2 ml to stimulate and record the nonlinear signal, as seen in Fig. 4b. The detailed drawing of electrodes can be seen in Fig. 4c and the electrodes are made of gold with no coating. The larger and external electrodes were used as driving, i.e., current injecting electrodes, while the smaller and internal electrodes were used for voltage recording.

### Electronic deriving circuit

The nonlinear dielectric spectrum was recorded after injecting an AC current of 20 mA and a frequency of 10 or 100 kHz into the solution and then recording the resulted voltage at the electrodes using a custom electronic module (Fig. 4a). We used the signal generator (AFG-2125 GW INSTEK) to generate sinusoidal voltage with an amplitude of 100 millivolts (V_in_ in Fig. 4). Initially, the harmonics of the device (which are non-biological and thus undesired) were characterized at different voltage levels by a spectrum analyzer (RIGOL DSA815-TG), and a voltage level of 100 mV at frequencies of 10 and 100 kHz was selected due to lower system harmonics seen in the output. This voltage level was then amplified by an operational amplifier (AD811) with a gain of 10 and fed into a floating current source. A constant current amplitude was established between the outer electrodes of the measurement chamber. The voltages of the inner electrodes were picked up by a differential amplifier, and the spectrum analyzer recorded the output harmonics (Fig. 4b).

There is a trade-off in choosing the input impedance of the differential amplifier between the electrode polarization effect and THD of the recording system. If the input impedance becomes less than a threshold, the polarization effect can disturb the recorded voltage. On the other hand, if it gets larger, the THD of the operational amplifier in the recording system will exceed the − 60 dB level, which is more than the required system THD threshold. The measured impedance of the virus solution was about 100 ohms, therefore, the value of 20 KOhms was chosen to be larger than the solution and still not too large to cause electrode polarization affect the readout, so that the best performance is achieved.

The electronic system plays a fundamental role in receiving the signal. For this reason, low harmonics and low noise was taken into consideration in the design of electronic circuit, printed circuit and selection of electronic components. The spectrum analyzer, a low-noise device with about − 100 dBm was used to increase the sensitivity and accuracy. Thorough attention was paid to the electrodes and chamber design to minimize their artefacts and intrinsic effects on the applied field and recorded signals. However, in order to characterize the system and detect possible Noise/Harmonic distortion, a series of separate tests were conducted before measuring the viral samples including: (i) Source Test, where Spectrum analyzer was connected directly to the signal generator and the harmonics of the signal generator were measured for values of frequency, output voltage and load comparable to actual measurement. The source harmonics were about − 60 dBc in our experiment. (ii) Resistance test, by which the overall harmonics of the system consisting of signal generator, measurement system and differential amplifiers (see Fig. 4) was determined. The added harmonics in this test, in which the electrodes were substituted by a set of resistors, whose resistance was comparable to that of the solution, were attributed to the nonlinearities of the differential amplifiers electronics. (iii) Solution test, where the recorded current of a known solution (DI water and/or Saline with comparable ionic strength placed in contact with the electrodes), revealed the electrode polarization related effects on the harmonics creation and its characterization.

## Results

### Theoretical results

**Figure 5:**
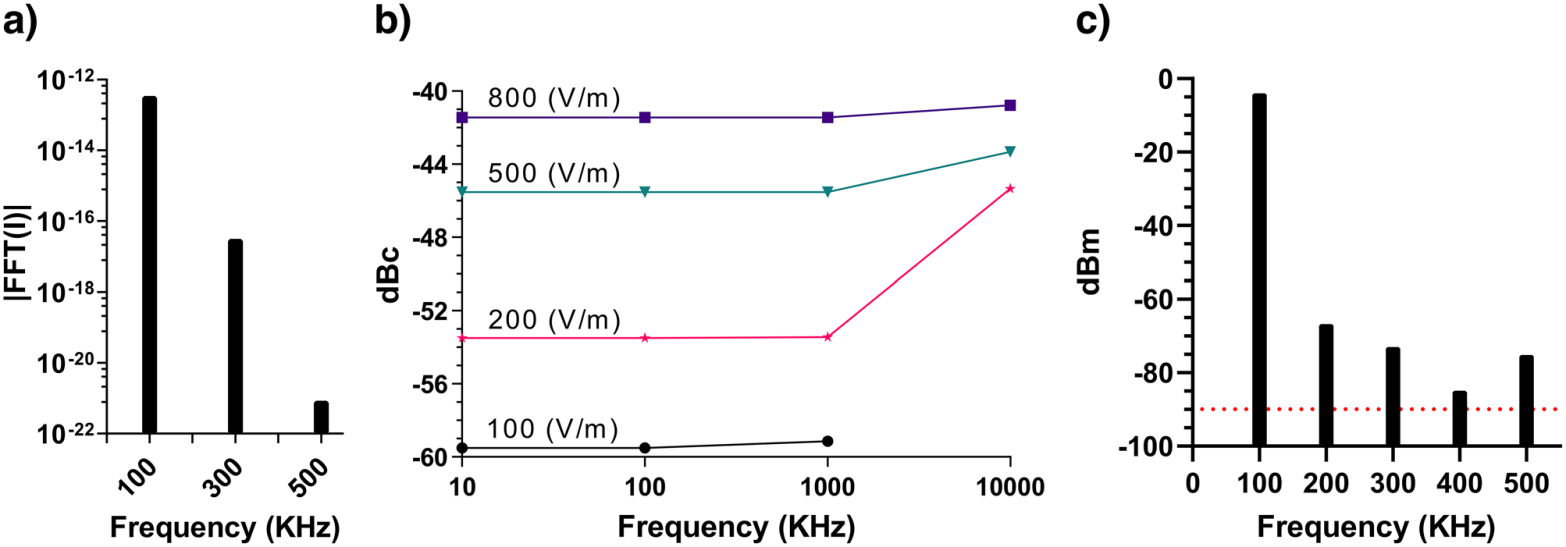
Simulated power spectrum and harmonics of virus and virus-free salt samples. (**a**) Simulated power spectrum of the voltage recorded from a virus suspension solution. (**b**) The ratio of the third harmonic to the main harmonic amplitudes versus frequency for different stimulation amplitudes. (**c**) Harmonics of the 280 mM NaCl solution. Drawings were made using Inkscape version 1.1 and GIMP version 2.10.28 (http://www.gimp.org, http://www.inkscape.org).

The simulation result for the presence of a virus between two electrode plates using the mathematical model for the ionic currents of the membrane is shown in Fig. 5a. In addition to the fundamental frequency, the odd harmonics also showed up in the power spectrum of the simulated voltage. Our goal was to find these harmonics and evaluate the ratio of their amplitudes to the main harmonic. Figure 5b illustrates the ratio of the third harmonic to the main harmonic, i.e., the fundamental excitation frequency, at different frequencies and with different excitation amplitudes. Also, measurement was conducted on a known saline solution, 280 mM NaCl, representing the level of overall residual system harmonics which was low enough for subsequent measurements of viral samples (Fig. 5c).

The ratio of the third harmonic to the main harmonic amplitudes depends on the frequency and the amplitude of the electric field stimulation. At higher frequencies and amplitudes, the presence of the third harmonic was stronger. The harmonic levels in this paper are given in units of dBc, which stands for dB relative to *carrier* (fundamental or main harmonic level). A third harmonic given as − 10 dBc is 10 dB *smaller* than the main harmonic. Theoretically, a third harmonic level of − 40 to − 60 dBc was estimated.

### Experimental results

**Figure 6:**
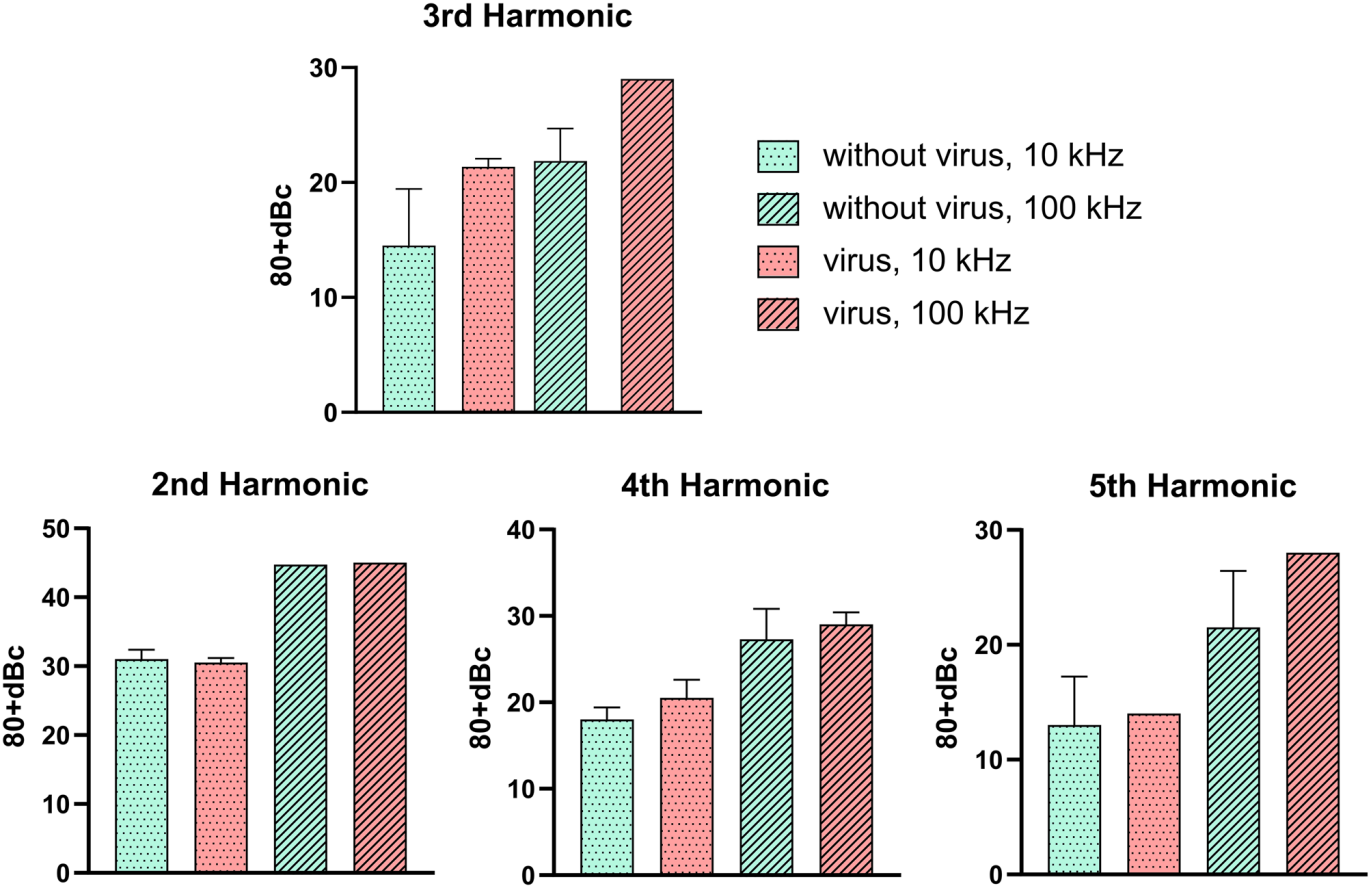
Experimentally measured harmonic levels. Drawings were made using Inkscape version 1.1 and GIMP version 2.10.28 (http://www.gimp.org, http://www.inkscape.org).

Harmonic measurement was performed for all patient samples, including positive and negative PCR labels. Each sample was measured twice. A 20 mA sinusoidal current was applied to the samples at 10 and 100 kHz frequencies. The resulting voltage signal between the sample and the reference solution was fed into the spectrum analyzer. The harmonic pattern was obtained, and the amplitudes of the measured harmonics were compared with the main harmonic in GraphPad Prism Software version 9.3.1 as reported in Fig. 6.

The sensitivity of the method presented here was based on the Ct value immerged from rtPCR. Accordingly, a rough idea of the number of viruses in the sample was worked out. The sensitivity of the method presented here, i.e., the signal strength per virus particle, was estimated as follows. The positive samples possessed a Ct value of at most 35 which was an indication of about 10^5^–10^7^ Viron/ml in the solution^17^. The positive samples had a third harmonic strength equal to approximately − 50 dBm (about 0.01 microwatts). This corresponds to a sensitivity of approx. 10^–14^ watts per virus particle. Also considering the 2 ml volume of the chamber, this results in a minimum concentration of the virus of about 10^4^/ml, that is needed to produce a reliable detection for our system which has a noise level of − 100 dBm. Under the conditions of our preliminary experiments, a rough estimation of accuracy is about $\pm 2 \;\text{dBc}$ for the third harmonic amplitude. This corresponds to about $\pm 5\times {10}^{5}$ viral particles which is reasonable for solutions containing the typical count of 10^6^ virions/ml or higher.

## Discussion

The theoretical results showed that the application of electrical stimulation caused a change in the membrane's voltage and resulted in nonlinear ion currents flowing in the membrane. This event was detected as a harmonic pattern in the voltage power spectrum recorded at the electrodes site.

The measurements involved several challenges and there are certain noise effects that have to be considered. In nonlinear spectroscopy, as for detecting the harmonic changes caused by the bio-solution, the undesired harmonics caused by the voltage source and the electronic measurement circuits shall be carefully analyzed and removed. The total harmonic distortion (THD) level should be less than 60 dB based on theory. Therefore, first, with a resistance test, i.e., using specific resistance elements instead of the bio-solution, the system and the source THD levels were measured, and the deriving circuit was carefully redesigned, and op-amps were reselected to have a THD level of less than 60 dB with high accuracy and validity in the absence of the bio-solution.

With the bio-solution in place, it is known that the polarization of the electrode in contact with the electrolyte has a nonlinear nature and could be considered as another significant source of undesired harmonics. Thus, a four-electrode system was used to eliminate this effect. In other words, based on Woodward's idea^11^, a reference solution is utilized to remove the nonlinear effect of the electrode. In this way, in addition to the patient sample solution, the harmonic spectrum was also recorded from the VTM reference solution, i.e., the virus-free solution. The common factors are eliminated by subtracting the reference signal from the sampled signal. The reason for choosing VTM is that it is a solution with electrode polarization harmonics close to positive and negative samples. By subtracting the VTM-only signal from the positive or negative virus containing sample signals, the virus-specific signal will present dominant harmonics. Furthermore, it is possible to set up two identical chambers, one including the sample solutions and the other as VTM-only. These two chambers can be connected by special membranes that allow the fluids to equilibrate in terms of ionic strength, pH, etc. without exchanging viral particles. The signals from these chambers can then be subtracted from each other to cancel non-virus harmonics.

As a result, the harmonic pattern obtained from the difference between these two signals is only due to the presence of the virus in the solution. It shall be emphasized that the accuracy of the detection depends on careful sample handling, thus to prevent damaging the virus or its RNA before performing the measurements.

Thus, it becomes possible to verify the appropriateness of the harmonics of the system and also to eliminate the harmonic effects of electrode polarization. Accordingly, we expect the positive samples to dominantly show harmonics caused by the viruses in the solution. We believe the critical precaution taken in the design, utilization and analysis of the results of the current approach and technique, paves the path for the definition and fabrication of the next generation techniques needed to detect various microorganisms.

In the recorded pattern, the third harmonic amplitude was strongly correlated with the presence of virus particles. The measured third harmonic level showed an overall agreement with the theoretically estimated level of − 60 to − 40 dBc. Furthermore, applying T-Test analysis on the values (pooled for all frequencies) showed that the positive and negative samples are distinguishable from each other significantly with a p-value of 0.018. Therefore, we believe our proposed NLDS technique has the capability of detecting the presence of SARS-CoV-2 particles.

This is the first time that nonlinear dielectric spectroscopy is being utilized towards the detection of viruses, in particular for the corona virus whose fast, cheap, and real time detection is vital. The method presented here is based on the electronic and electrical aspects of the microorganism studied and does not require any sample preparation and labeling nor relies on the expensive chemical and/or molecular ingredients used in routine methods such as rtPCR. Furthermore, possibilities of tailoring the method to detect in a fast, real time and portable manner at low expenses indicates its superiority over current detection methods. Another newly-emerging technology to detect pathogenic microorganisms, is based on electrochemical sensing of Reactive oxygen species (ROS) released by microorganisms^18^. This method interacts with released material from the microorganisms, whereas the proposed method here interacts directly with microorganism structure. The promising preliminary results paves the path and encourages our ongoing research to improve its specific sensitivity, accuracy and reliability not only for the detection of viruses but also to distinguish between different strains of viruses and also other microorganisms in future.

The concentration of the virus copies in the sample was worked out based on the cycle threshold value (known as Ct as well as, aka Cq, or Cp) that emerged from the RT-PCR tests that depends on different factors considered and applied at test centers. The positive samples possessed a Ct value of at most 35 which was an indication of about 10^5^–10^7^ Viron/ml in the solution^17^. At low concentrations, the relationship between the harmonic amplitude and the virus concentration was expected to be linear^11^. Accordingly, for a 10 time increase in the number of viruses in the solution, the harmonic level would increase by 20 dB and vice versa. The most nonlinear effect was caused when high number of viruses were present on the surface of the electrodes due to the stronger electric field. Thus, at high concentrations, the surface of the electrodes would saturate with virus particles and the harmonic strength does not increase with increasing viral content. Further to the RT-PCR, other techniques such as Dynamic Light Scattering (DLS) and Quantal Assays, TCID50, LD50, EID50 are being used to identify the actual number of the viruses in the sample, with less than 50% accuracy.

Phase information is also useful for the analysis of impedance spectra of biological systems^19^. Therefore, more extensive data can possibly be obtained by using a digital lock-in amplifier or phase-sensitive signal analyzer as the output device.

We now turn our attention from technical aspects to a short discussion of the fundamental mechanisms: Membrane proteins possess significant dielectric moments^20^ that makes them susceptible to external electric fields. The amplitude and frequency of the external field contribute to major electrostatic forces on the membrane proteins. The stationary status of the proteins in the membrane hiders their conformation change or rotation across membrane to dissipate the imposed electrical energy by simple Debye-like rotation^11^ causing a signature response that is manifested by the intensity, frequency and/or harmonics of the resulting signal. The membrane is excited by the applied field and the dielectric moment of the membrane proteins together with membrane surface potential caused by charges of both membrane constituent lipids and proteins as well as the dispersion and relaxation of the counter ions arranged tangential to the membrane surface in the surrounding electrolyte indicate the significant generation of a non-linear dielectricity^21^. The arrangement that is manifested by electric double layers (EDL) and potentials such as zeta potential, make a unique bioelectrical status that is deviated by the applied field and responds in an exceptional manner. The spatial and temporal diversity of the distribution, arrangement and dynamics of the membrane constituent polyelectrolyte molecules as well as surrounding charges makes the rather dynamic and complex bioelectric and bioelectronics profile of system that responds in a nonlinear manner to the frequency, intensity and harmonies of the applied signals.

Based on the resulting entropy level, and the odd or even harmonic produced one might work out the amount of the energy spent to change the conformation of the channel, membrane part of the spike as the major source of the resulted effect on the signal^11^, as well as the membrane activation and rearrangement of the counter ions on the membrane surface. These mechanisms are well known. But our paper suggests a novel mechanism for the harmonics in SARS-Cov-2, which is the ion channel on the viral membrane with nonlinear IV characteristic. The overall agreement obtained between a mathematical model based on this assumption and our experimental results might support this mechanism. More detailed biomolecular studies are necessary, however, to understand the real mechanism(s) involved. We suggest these as future work in this next-generation technology.

Finally, our experimental observations supported a third harmonic growth in solutions with positive PCR tests compared to the negative PCR tests at both selected frequencies of 10 and 100 kHz. The presence and activity of the Orf3a ion channel in the SARS-CoV-2 virus membrane is hypothesized to be the main source of harmonic generation. Accordingly, our NLDS-based detection system might be further developed at different pH and ionic conditions to distinguish between different virus strains. The method presented here is being further developed to differentiate between virus strains. As the bioelectric and bioelectronics of individual strains of viruses are dependent on the pH and ionic strength, these factors clearly play a major role. Different measures can be extracted from the data. For example, the ratios between harmonics can be one way to eliminate common factors (such as ionic strength and pH) that influence every harmonic amplitude. Accordingly, we consider that absolute harmonic amplitudes are affected by various factors including, (i) ionic strength and counter ion type and concentration (ii) pH of the virus containing medium, (iii) temperature, (iv) surface charges of the virus formed by its membrane lipids, proteins and peripheral sugars and/or linking molecules, (v) ionic permeability through either channels or lipid layers in the first place. Thus, in order to tailor an efficient method to detect viruses or other microorganisms, relative measures, such as the ratio between the third and first harmonics (h3/h1) should be considered. We anticipate that harmonic ratios tend to cancel out the common effects of the medium ionic strength and conductivity, and be more sensitive to biological nonlinearities. Such measures can be obtained experimentally for different virus strains under various pH and ionic strength conditions. Subsequently classification, and pattern recognition techniques can be used to define criteria for distinguishing different viruses, as the next generation techniques used to detect different microorganisms.

## Conclusion

The worldwide prevalence of COVID-19 disease and the need for early and PoC type diagnosis have led to numerous efforts in this area through inventing new techniques for virus detection. In this study, changes in the third harmonic pattern of the recorded power spectrum resulting from electrical excitation of the patient sample solution are introduced as new tentative biomarkers for virus detection. It was demonstrated that the third to first harmonic amplitude ratio can be significantly different between positive and negative samples. This difference is promising as it can potentially be used as a diagnostic tool. Using this biomarker alone or in combination with other methods, like electrochemical methods or through electrode surface functionalization for virus-specific binding, we can conveniently and efficiently improve the SARS-CoV-2 detection.

We strongly believe that the proposed NLDS technique, by careful choice of the optimal frequency and the voltage range to detect the presence of the virus in a patient sample, regardless of its genetic content, could open a new avenue in designing SARS-CoV-2 diagnostic kits. Although the setup presented here still needs further development in order to be used as a stand-alone diagnostic kit, we hope the outcomes of our ongoing experiments will form a basis for the detection of all pathogenic microorganisms, including different kinds of viral agents, with high accuracy in near future.

## Supplementary Information

Supplementary Table S1.

## Data Availability

All data generated or analyzed during this study are included in this published article and its supplementary information files. See Supplement 1.docx for tables of harmonic readouts.
