# Supplementary material for: Nonlinear dielectric spectroscopy biosensor for SARS-CoV-2 detection
Source: Sci Rep. 2022 Oct 12;12:17080. doi: 10.1038/s41598-022-20961-7 (PMC9554844; doi:10.1038/s41598-022-20961-7)
Supplement: Supplementary file 1 — Supplementary Table S1. [file 41598_2022_20961_MOESM1_ESM.docx]

**Supplementary Material**

for the paper *Nonlinear Dielectric Spectroscopy Biosensor for SARS-CoV-2 Detection* by Ali Talebipour et al.

| Table S1. Experimentally measured harmonic levels. | | | | | | |
| --- | --- | --- | --- | --- | --- | --- |
| **PCR result** | **Trial number** | **Frequency** | **2^nd^ harmonic (dBc)** | **3^rd^ harmonic (dBc)** | **4^th^ harmonic (dBc)** | **5^th^ harmonic (dBc)** |
| Negative | First | 100 kHz | -35 | -56 | -55 | -55 |
| Positive | First | 100 kHz | -35 | -51 | -50 | -52 |
| Negative | Second | 100 kHz | -35 | -60 | -50 | -62 |
| Positive | Second | 100 kHz | -35 | -51 | -52 | -52 |
| Negative | First | 10 kHz | -48 | -69 | -63 | -64 |
| Positive | First | 10 kHz | -49 | -59 | -58 | -66 |
| Negative | Second | 10 kHz | -50 | -62 | -61 | -70 |
| Positive | Second | 10 kHz | -50 | -58 | -61 | -66 |

**dBc=dB relative to Carrier Frequency Power (1^st^ Harmonic)**
